# Periprocedural Myocardial Infarction following Elective Percutaneous Coronary Interventions

**DOI:** 10.31083/j.rcm2309309

**Published:** 2022-09-13

**Authors:** Antonio Landi, Claudio Montalto, Gabriele Crimi, Stefano De Servi

**Affiliations:** ^1^Division of Cardiology, Cardiocentro Ticino Institute, Ente Ospedaliero Cantonale (EOC), 6900 Lugano, Switzerland; ^2^Cardiologia 1 – Emodinamica, De Gasperis Cardio Center, ASST Grande Ospedale Metropolitano Niguarda, 20162 Milano, Italy; ^3^Interventional Cardiology Unit, Cardio-Thoraco Vascular Department (DICATOV), IRCCS Ospedale Policlinico San Martino, 16132 Genova, Italy; ^4^Department of Molecular Medicine, University of Pavia Medical School, 27100 Pavia, Italy

**Keywords:** periprocedural myocardial infarction, chronic coronary syndrome, percutaneous coronary intervention

## Abstract

The prognostic relevance of periprocedural myocardial infarction (PMI) in patients with chronic coronary syndrome undergoing percutaneous coronary intervention (PCI) is still matter of debate, particularly regarding the type (cardiac troponin or creatin kinase-MB) and different thresholds of biomarkers elevation, as the importance of associated ancillary criteria of ischemia or concomitant angiographic complications. There are still uncertainties regarding the value of PMI as event which is prognostically equivalent to spontaneous myocardial infarction or if it simply represents a marker of baseline risk, atherosclerotic burden and procedural complexity. In the present review, we will present the mechanisms and predictors of PMI occurring during PCI and potential treatment strategies to reduce its occurrence. We will also overview all commonly adopted definitions of PMI, which carry different prevalence and prognostic implications in daily practice and clinical trials. Finally, we will discuss the impact of different PMI definitions on the interpretation of trials results, emphasizing the importance of adequate endpoints selection in the planning and interpretation of clinical trials.

## 1. Introduction

Percutaneous coronary interventions (PCI) is a widely used revascularization modality for patients with chronic coronary syndromes (CCS). Although technical advances and new pharmacological therapies have drastically reduced PCI-related complications such as acute stent thrombosis or access site bleeding events, post-PCI increases in cardiac biomarkers are frequent, especially if high-sensitivity cardiac troponins (hs-cTn) are systematically measured after the procedures [1]. The prognostic relevance of such elevations in terms of recurrent cardiovascular events and long-term mortality is still debated: particularly it is unclear whether the definition of peri-procedural myocardial infarction (PMI) should be based on the isolated finding of biomarkers elevations or if the definition is fulfilled only when the concomitant association of new ischemia or documented angiographic complications are present [2, 3]. The controversy also concerns the type of cardiac biomarkers to be measured, whether creatine kinase-MB (CK-MB) or hs-cTn [4, 5]. Moreover, although it is mostly agreed that the definition of PMI should bear a relationship with early and late mortality, it is controversial whether this association is causal or simply reflects the prognostic relevance of more severe atherosclerosis [6, 7].

Multiple review and consensus papers have addressed the value of different definitions of PMI in patients undergoing elective PCI. However, evidence continues to evolve, and a single updated document is still missing. In the present review, we discuss the mechanisms and predictors of PMI as potential treatment strategies to reduce its occurrence. We will also overview all commonly adopted definitions of PMI, discussing the impact of different definitions on the interpretation of trials results.

## 2. Mechanisms of Periprocedural Myonecrosis and the Risk of Confounding

**Fig. 1.**
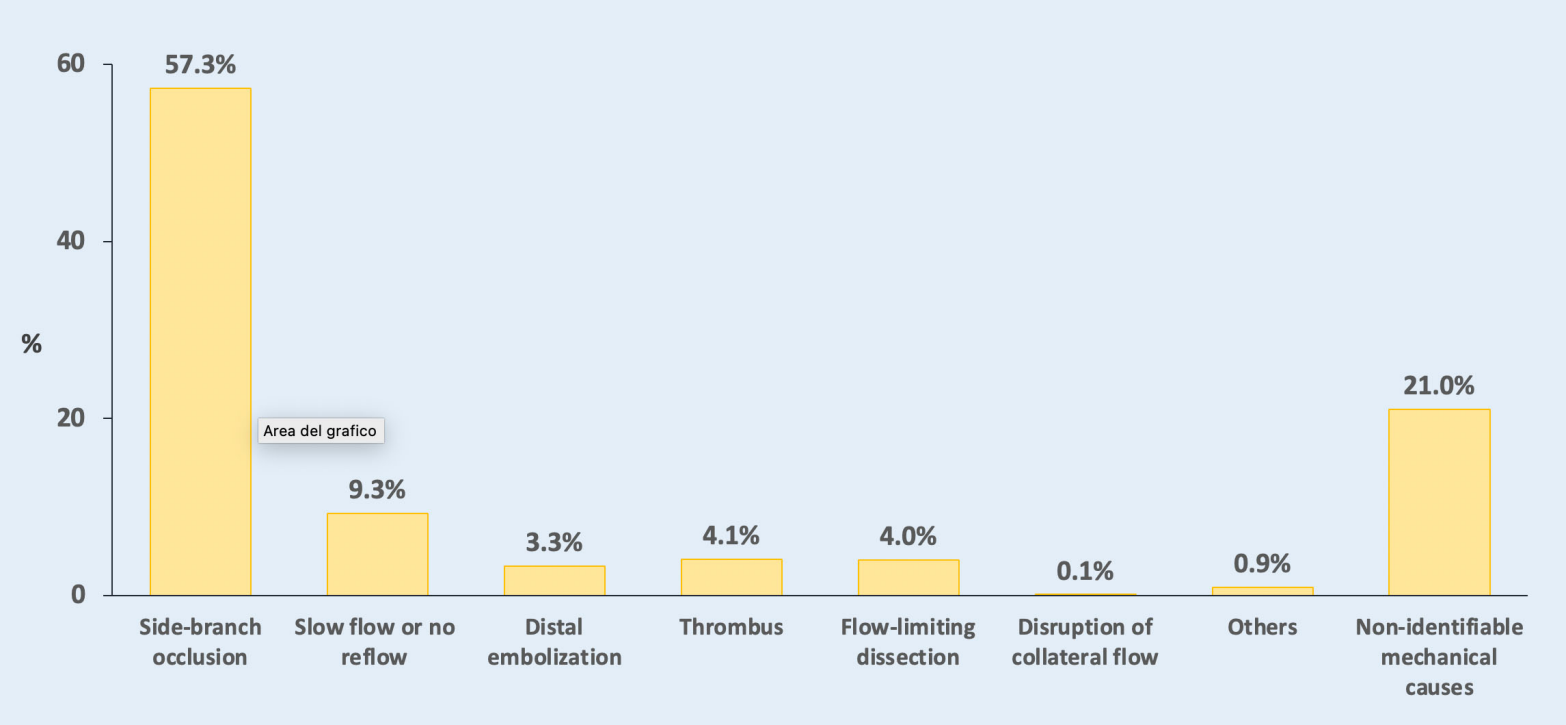
**Main causes of periprocedural myocardial infarction**.

Angiographically evident complications may occur during PCI, such as occlusion of side branches, flow-limiting coronary artery dissection, distal embolization of plaque components including thrombus, platelets or atherosclerotic debris, intraprocedural stent thrombosis, disruption of collateral flow, and slow-/no-reflow phenomena [8]. All these conditions may lead to myocardial injury that can be detected by cTn elevations, with different prevalence as shown in Fig. 1 [9]. Although large PMI are usually secondary to angiographically visible complications after PCI, in the vast majority of patients with elevated biomarker levels there is no evidence of procedural complications [10]. As shown by cardiac magnetic resonance studies, there are two distinct sites for procedural myonecrosis, with a mean infarct size of approximately 5% of the left ventricular mass, equally occurring at the two locations: the first is adjacent to the site of the intervention, likely secondary to epicardial side-branch occlusion, whereas the second is downstream from the intervention site, possibly related to the impairment of the microvascular circulation [11].

Distal embolization of thrombus and/or plaque debris released by balloon inflation, particularly when stents are implanted, can be detected as high-intensity transient signals using intracoronary Doppler guide wire [12]. The composition of the plaque affects the occurrence and the extent of periprocedural necrosis: plaques with large necrotic cores can cause greater degrees of myonecrosis, whereas fibrous plaques are relatively inert in this regard [13]. A large overlap of the magnitude of plaque microembolization between patients with and those without PMI has been observed suggesting that other factors, such as release of vasoactive substances, platelet activation, and vulnerability of the myocardium play a role in the pathogenesis of PMI [14].

A series of factors are significant predictors of PMI [9]. Among the clinical variables, advanced age, female gender, diabetes mellitus, hypertension and renal dysfunction were found to be significantly associated with PMI. Multivessel coronary artery disease (CAD), left anterior descending or left main disease, bifurcations, long lesions, drug-eluting stents (DES) and number of DES were also independent angiographic predictors of PMI. Interestingly, cTn release after elective PCI also correlates significantly with lesion complexity and extent of coronary artery disease, as measured with the Syntax score [15], suggesting that cTn release after PCI may be simply a marker of baseline risk, atherosclerotic burden and procedural complexity. These data call for caution in considering “stand alone” biomarkers elevations as indicative of procedural complications or to use them as a quality metric for PCI. To this regard, data from the National Cardiovascular Data Registry showed that hospitals routinely performing marker testing after PCI had higher rates of PMI detection despite a trend toward lower mortality and greater adherence to recommended medications that suggest better overall quality of care for PCI patients at those hospitals [9].

## 3. The Definition of PMI: A Historical Perspective

Although enzyme elevations after PCI were first reported in the early times of coronary balloon angioplasty [16], they were thought to be common findings associated with the procedure, not resulting in permanent clinical sequelae [17]. It was only in mid-90’s, however, that a significant relationship between such elevations and subsequent mortality was recognized. In analysis of 4664 consecutive patients who underwent successful coronary angioplasty or directional atherectomy at the Cleveland Clinic, Abdelmeguid *et al*. [18] found that progressive increases in CK-MB after the procedure were associated with an increasing number of procedural complications, such as coronary embolism, transient in-laboratory closure, hemodynamic instability and large dissections. After a mean follow-up of 3 years, cardiac-enzyme elevation was an important correlate of cardiac death (risk ratio: 2.19), without differences between CK-MB increase above 2 or 5 times control values. The authors concluded that CK elevations after PCI as low as twice the upper CK limit of normal (ULN) has a significant impact on long-term outcomes. Data from The Evaluation of Platelet IIb/IIIa Inhibition for Prevention of Ischemic Complication (EPIC) trial confirmed those findings, showing that even relatively minor degrees of CK elevation were associated with adverse events, even though it was recognized that the higher the rise in CK-MB, the greater the impact on prognosis [19].

The consistency of these findings led to the widespread acceptance of a negative role for PMI and moderate elevations of CK-MB (>3 times the ULN) were considered as appropriate surrogate endpoints for studies of coronary interventional devices and antithrombotic drug strategies [7].

With the advent of sensitive troponin measurements, being capable of detecting smaller amount of myocardial necrosis than CK-MB, it was found that at least 50% of patients undergoing PCI had post-procedural cTn elevations and that this biomarker was oversensitive in diagnosing PMI. With cTn, at the same thresholds as those used for CK-MB (3–5× ULN), the prevalence of PCI-related MI is substantially higher, but indicates a smaller amount of cellular damage, with uncertain clinical consequences. Moreover, only a small minority of patients had evidence of cardiac magnetic resonance abnormality on late gadolinium when a cTn threshold 3× ULN was used [20]. Tricoci *et al*. [21] found that the mortality risk associated with CK-MB of 3 or more ULN was reached by cTn at a threshold of approximately 60× ULN in patients with non-ST elevation MI. When a CK-MB threshold of >5× ULN was considered, a cTn >100× ULN was needed to have a similar mortality risk. The proportion of patients who had values above these thresholds was similar suggesting that the extent of myocardial damage may be comparable at those thresholds. Novack *et al*. [22] showed that a cTn of >20× ULN provided a comparable risk of 1-year mortality and similar frequency of MI as CK-MB >3× ULN in elective PCI patients. Although the cTn to CK-MB equivalency threshold varied in the two studies, probably due to the different clinical presentation of their respective populations (acute coronary syndrome versus elective patients) the findings are similar showing that both cTn and CK-MB elevation following PCI are associated with mortality, but the threshold is much higher for cTn than for CK-MB.

Several studies, mostly retrospective, have been conducted to assess the association of post-PCI cTn increase with prognosis, with very inconsistent results. Cavallini *et al*. [23], in a prospective study, showed that isolated cTn increases have a more questionable prognostic significance than CK-MB increases. The authors found that only CK-MB—not cTnI—was associated with increased mortality at a mean follow-up period of 2 years, confirming their results in a further analysis restricted to patients with baseline normal CK-MB and cTnI values and without CK-MB elevation after PCI [24]. Interestingly, a cTnI elevation >0.45 ng/mL was associated with a rise in 2-year mortality which, however, did not remain significant after controlling for concomitant risk factors (age, diabetes, renal insufficiency, peripheral arterial disease, multivessel disease, and left ventricular ejection fraction) in the multivariable analysis.

Although it is expected that the higher mortality risk of PMI is dependent on the defined values of cardiac biomarkers increase (e.g., higher values, worse prognosis), establishing higher cut-off could lead to overlook patients with mildly or moderately increased cardiac enzyme dying long time after PMI. Therefore, any definition for predicting the trade-off between short and long-term mortality risks should take into account the patient population, the PMI definitions used, the duration of the observation period, and the timing of risk assessment.

## 4. Universal Definition of Myocardial Infarction, Academic Research Consortium and Society for Cardiovascular Angiography and Interventions definitions of PMI

Despite the lack of any clear evidence, the 2007 universal definition of myocardial infarction (UDMI) defined as type 4a MI (that is MI following PCI) any increase of myocardial necrosis biomarker, either cTn or CK-MB, above three times their respective upper reference limit (URL) [25]. The basis for that decision was that there was no solid scientific basis for defining a biomarker threshold for the diagnosis of PMI, therefore, by arbitrary convention, any increase more than three times the 99th percentile URL was considered indicative of PCI-related myocardial infarction (type 4a). This drastic position was mitigated in the latest versions—the third and fourth UDMI [26, 27]—in which Type 4a MI requires an increase of cTn values >5 times the URL in patients with in-range cTn at baseline or a 20% elevation in those with high but stable pre-PCI cTn levels, associated with “ancillary criteria”, such as evidence of new myocardial ischaemia, either from ECG changes, imaging or from procedural complications causing reduced coronary blood flow (coronary dissection, occlusion of a major epicardial artery or a side branch occlusion/ thrombus, disruption of collateral flow, slow flow or no-reflow, or distal embolization). “Stand-alone” post-procedural increases of cTn values were considered necessary to establish a diagnosis of “procedural myocardial injury” but not for the diagnosis of type 4a MI. The only evidence quoted in that document to support that definition was an analysis of 1390 PCI patients with negative baseline cTn levels, showing that patients with PMI, defined according to the third UDMI, had a higher number of ischemic events, defined as cardiovascular death, MI, ischaemic stroke, and refractory angina [1]. The statistically significant difference between the two groups was confirmed in a multivariable analysis. There were however large clinical and angiographic differences among patients with and without PMI, with a higher risk profile in the PMI group. Five variables were significantly related to the occurrence of PMI: older (>75 years) patients, glomerular filtration rate (GFR) <60 mL/min, left main disease, stent length ≥30 mm, number of stents ≥3, but only age was included in the multivariable analysis of 1-year ischemic events.

In 2013, an expert consensus document was issued from the SCAI to challenge the PMI definition proposed by the task force of the UDMI [28]. The authors of the document argued that the criteria used to define type 4a MI were arbitrarily chosen, of debatable clinical relevance and not grounded on substantial scientific evidence. Not only did the authors question the arbitrary cut-off of cTn to 5× URL, but also the relevance of the “ancillary criteria”, since angiographically evident complications are not always associated with sizable post-PCI biomarker elevations, and biomarker elevations can occur without angiographic complications. Since a threshold post-PCI level of cTn above which long-term prognosis is affected has not been established, in that document CK-MB was strongly preferred to assess clinically relevant post-PCI MI events. A clinically relevant MI occurring in the post-PCI period was defined as that resulting in a CK-MB >10× ULN or in a lower threshold (>5× ULN) in patients in whom new pathologic Q-waves in >2 contiguous leads (or new persistent left bundle branch block) develop post-PCI.

The ARC-2 revised the definitions of clinical and angiographic endpoints in coronary device trials, initially proposed in 2007, to standardize their use in clinical trials that frequently include complex patient populations and lesions [29]. The authors observed that in recent years cTn has been progressively substituted by CK-MB as the preferred biomarker of myocardial injury in clinical practice and proposes a ≥35 URL threshold for PCI-related PMI as a reasonable threshold. One ancillary criterion was also required in addition to the ≥35 cTn absolute rise to fulfil the definition of PMI (“flow-limiting” angiographic complications in a major epicardial vessel or >1.5-mm diameter branch, new significant Q waves (or equivalent) related to the procedure, or a substantial new wall motion abnormality on echocardiography related to the procedure). A significant periprocedural myocardial injury was defined as a rise in cTn ≥70 times the URL, in the absence of ancillary criteria. The different definitions for PMI are summarized in Table 1.

**Table 1.** **Definitions of periprocedural myocardial infarction according to the fourth UDMI, ARC-2 and SCAI**.

4th UDMI	cTn >5 times the 99th percentile URL AND one of the following additional elements: (1) New ischaemic ECG changes; (2) Development of new pathological Q waves; (3) Imaging evidence of new loss of viable myocardium or new regional wall motion abnormality in a pattern consistent with an ischaemic aetiology; (4) Angiographic findings consistent with a procedural flow-limiting complication such as coronary dissection, occlusion of a major epicardial artery or a side branch occlusion/thrombus, disruption of collateral flow, or distal embolization.
ARC-2	cTn ≥35 times URL AND one of the following criteria: (1) New significant Q waves or equivalent, (2) Flow-limiting angiographic complications (loss of patency of major vessel, graft, or side branch; embolization; Disruption of collateral flow; persistent slow flow or no reflow; major dissection; coronary artery bypass graft surgery specific); (3) New “substantial” loss of myocardium on imaging.
SCAI	In patients with normal baseline cTn: cTn ≥70× ULN, or ≥35× ULN with new pathologic Q-waves in ≥2 contiguous leads or new persistent LBBB.
	In patients with elevated baseline cTn in whom the biomarker levels are stable or falling: the cTn rises by an absolute increment equal to those levels recommended above (from the most recent pre-procedure level) plus new ST-segment elevation or depression plus signs consistent with a clinically relevant MI, such as new onset or worsening heart failure or sustained hypotension.

Abbreviations: MI, myocardial infarction; UDMI, universal definition of myocardial infarction; ARC-2, Academic Research Consortium-2; SCAI, Society for Cardiac Angiography and Interventions; URL, upper reference limit; ULN, upper limit of normal.

**Fig. 2.**
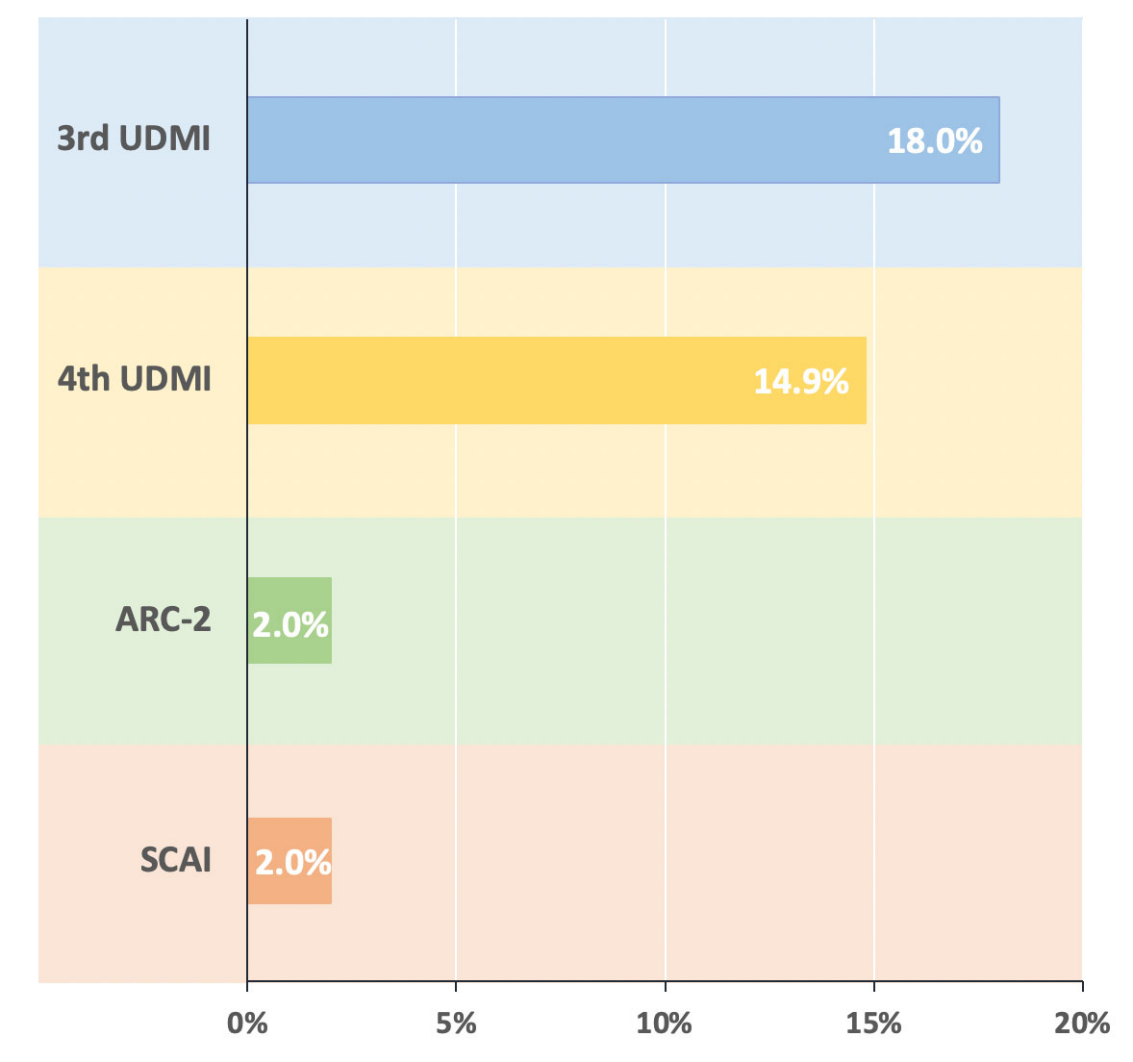
**Different rates of periprocedural myocardial infarction in chronic coronary syndromes patients undergoing PCI according to the various definitions used**. Abbreviations: UDMI, universal definition of myocardial infarction; ARC-2, Academic Research Consortium-2; SCAI, Society for Cardiac Angiography and Interventions.

The incidence of PMI greatly varies according to the different criteria used for the diagnosis. Recent literature data showed that, among 4404 patients with CCS undergoing PCI [30], PMI defined by the third UDMI, fourth UDMI, ARC-2, and SCAI were observed in 18.0%, 14.9%, 2.0%, and 2.0% of patients, respectively (Fig. 2).

## 5. The European Society of Cardiology (ESC) Consensus Document on Prognostically Relevant Peri-Procedural Myocardial Injury and Infarction 

A recent Consensus Document endorsed by the ESC recommends the measurement of baseline and post-PCI cTn values in all CCS patients treated with PCI [31]. The document endorses the definition of Type 4a MI (post-PCI cTn elevation >5-fold the URL associated with the “ancillary criteria” of peri‑procedural angiographic flow-limiting complications, electrocardiographic or imaging evidence of new myocardial ischaemia) proposed by the task force of the UDMI, also stating that a similar increase in cTn , even in the absence of those ancillary criteria (a condition called major peri‑procedural myocardial injury) has prognostic relevance and its incidence after a PCI procedure should be used as a “quality metric and surrogate endpoint for clinical trials” [31].

These conclusions are mainly based on an individual patients data meta-analysis performed by Silvain *et al*. [32], that included 9081 stable patients undergoing PCI focused on post-PCI cTn elevations. In that study, the incidence of type 4a MI, according to the fourth UDMI, was a strong independent predictor of 1-year all-cause mortality. Major peri‑procedural myocardial injury was also independently related to 1-year all-cause mortality. One important limitation of that analysis is that the evidence of procedural complications and/or new myocardial ischaemia was available in about 25% of patients (n = 2316). Therefore, only in that limited subset of patients it was possible to ascertain the presence of type 4a MI, according to the URL (diagnosed in 12.7% of patients). Moreover, the multivariate analysis including those 2316 patients failed to show any independent relationship to 1-year all-cause mortality for major peri-procedural myocardial injury whereas type 4a MI was found to be a significant predictor (adjusted odds ratio 3.21, 95% confidence interval (CI): 1.42–7.27) [32]. Major periprocedural myocardial injury, diagnosed in 18.2% of patients, was prognostically relevant in the overall population, but in most patients type 4a MI could not be assessed, raising the doubt that many major peri‑procedural myocardial injury cases were indeed unrecognized type 4a MI and that the significant relationship observed in the multivariable analysis was secondary to undiagnosed Type 4a MI [33].

It must be observed that the pathophysiology of cardiac biomarkers release following PCI is multifactorial in aetiology. As already discussed, a strong association was found between post-procedural enzymes rise and large atherosclerotic plaque burden, coronary calcifications and lesion types, as detected by angiography and intravascular ultrasound imaging [34, 35]. Since the extent and complexity of coronary atherosclerosis is an independent predictor of mortality after PCI [36, 37], the association between biomarkers elevation after PCI and mortality may be simply an epiphenomenon, not due to a causal relationship [5, 28]; therefore multivariable analyses should incorporate all clinical and angiographic variables affecting outcome to determine whether the biomarker elevation is an independent correlate of mortality.

Further objections to the document concern the absence of comparative data on prognostic information with alternative PMI definitions such as those proposed by ARC-2 and SCAI [38]. Recently, Ueki *et al*. [30] assessed PMI according to the third and fourth UDMI, ARC-2 and SCAI criteria based on high-sensitivity cTn in patients with stable coronary disease treated with PCI enrolled into the Bern PCI registry. This analysis represents the first detailed evaluation of contemporary PMI definitions in elective PCI patients based on systematic hs-cTn measurements and systematic assessment of ancillary criteria in a large real-world PCI population. The primary endpoint was cardiac death at 1 year. Among patients with PMI, 1-year cardiac mortality according to the third UDMI, fourth UDMI, ARC-2, and SCAI, was 2.9%, 3.0%, 5.8%, and 10.0%. The ARC-2 (HR: 3.90; 95% CI: 1.54–9.93) and SCAI (HR: 7.66; 95% CI: 3.64–16.11) were more relevant compared with the third UDMI (HR: 1.76; 95% CI: 1.04–3.00) and fourth UDMI (HR: 1.93; 95% CI: 1.11–3.37) for cardiac death at 1 year.

Interestingly, the authors did not confirm the relationship between PMI defined by the fourth UDMI and all-cause mortality shown by Silvain *et al*. [24]. Likewise, no independent association was observed between major myocardial injury and cardiac death at 1 year. The conclusion was that PMI defined using the ARC-2 and SCAI criteria are more prognostic for cardiac mortality at 1 year compared with the third and fourth UDMI. Therefore, the ARC-2 and SCAI definitions may be preferred in daily practice and clinical trials [30].

## 6. Relevance of Various PMI Definitions to Results of Clinical Trials 

PMI is a component of primary or secondary endpoints of clinical trials evaluating the treatment effect of coronary devices and drugs in patients with coronary artery disease. Major myocardial injury is also frequently included into composite endpoints to achieve powered sample size, thus artificially affecting the interpretation of the real benefit of a treatment. The rates of PMI are highly dependent on their definitions, with dramatic variations of trials results using differing definitions. As an example, in the SYNTAXES (*Synergy between PCI with Taxus and Cardiac Surgery Extended Survival*) trial, in which patients with 3-vessel disease and/or left main coronary artery disease were randomized to undergo either PCI or coronary bypass grafting (CABG) surgery [39], the rates of PMI according to the fourth UDMI were 3.0% and 2.1% for PCI and CABG, respectively. Conversely, according to the SCAI definition, the rates were higher for both revascularization procedures (PCI 5.7% and CABG 16.5%). As a result, 5-year rates of major adverse cardiac and cerebrovascular events were lower for CABG versus PCI patients using the fourth UDMI (25.8% versus 37.5%) but similar (36.6% versus 38.4%) using the SCAI definition [39]. Analogous data were found in the EXCEL (*Evaluation of XIENCE versus Coronary Artery Bypass Surgery for Effectiveness of Left Main Revascularization*) trial in which patients with left main coronary artery disease were randomized to PCI versus CABG. In the intention-to-treat population, the rates of the primary endpoint (the composite of death, all MI, or stroke) were similar after PCI and CABG at 3 and 5 years when using the pre-specified protocol definition of PMI (similar to the SCAI definition), but were greater after PCI compared with CABG when PMI was determined by the third UDMI [40].

The relevance of PMI definition issue is also shown in the interpretation of the results of the *International Study of Comparative Health Effectiveness with Medical and Invasive Approaches* (*ISCHEMIA*) trial, which randomly assigned patients with stable CAD and moderate-to-severe myocardial ischemia to an initial invasive or conservative strategy [41]. In that trial, MI events were the predominant component of the primary and major secondary outcomes. Two definitions of PMI were adopted, one based on CK-MB rise >5-fold the URL associated with additional electrocardiographic and angiographic criteria for new ischemia or flow-limiting complications (primary definition), whereas the secondary definition relied on cTn rise >5-fold the URL associated with the above-mentioned additional criteria. At odds with the type 4a MI fourth UDMI, in the ISCHEMIA trial PMI could also be determined in the absence of such clinical, electrocardiographic, and imaging criteria (“stand-alone MI”) if CK-MB raised >10-fold the URL (primary definition) or cTn raised >70-fold the URL (secondary definition). As for the SYNTAXES and EXCEL trials, these findings were greatly affected by the definition used: with the primary MI definition, there were no major long-term differences in the primary composite outcome, whereas with the secondary definition a significant difference in the primary trial endpoint in favour of the conservative strategy was found [41].

A key point in the discussion about the most appropriate definition of PMI revolves around its impact on subsequent cardiovascular death. Since PMIs are added to spontaneous (type 1) MIs in the computation of long-term events it seems reasonable to use a definition of PMI that has a similar effect on cardiovascular mortality as that caused by type 1 MI [42, 43]. In this context, the data reported from ISCHEMIA trial provide interesting insights [44]. When primary and secondary definitions were applied to Type 1 MI, numbers of patients with this event were similar (217 and 223 cases) whereas using the secondary definition for PMI, number of events were more than twice as high as those determined using the primary definition (Table 2). Type 1 MI rates were higher in the conservative than in the invasive strategy group and were associated with all-cause and cardiovascular mortality using both primary and secondary definitions. On the contrary, PMI rates using both definitions (globally reported for PCI and CABG interventions) prevailed in the invasive group, but were not significantly associated with all-cause mortality and cardiovascular mortality [44]. Excluding from the analysis “stand-alone MIs”, PMI was significantly associated with cardiovascular mortality using the primary definition, showing that isolated cardiac biomarkers release may be clinically irrelevant and artificial in the assessment of otherwise robust composite endpoints [42, 43].

**Table 2.** **Relationship between spontaneous (type 1) and periprocedural myocardial infarction (MI) with cardiovascular mortality in the ISCHEMIA trial**.

MI type	Number	HR (95% CI)
		MI vs no-MI
PRIMARY DEFINITION		
Type 1 MI	217	3.38 (2.03–5.61)
Procedural MI	89*	1.99 (0.73–5.43)
Procedural MI (excluding stand-alone MI)	${\mathrm{36}}^{\#}$	3.75 (1.17–11.97)
SECONDARY DEFINITION		
Type 1 MI	223	3.52 (2.11–5.88)
Procedural MI	${\mathrm{245}}^{§}$	1.24 (0.57–2.68)
Procedural MI (excluding stand-alone MI)	115^	1.95 (0.79–4.84)

Multivariable models included the following variables: age at randomization, sex, estimated glomerular filtration rate, left ventricular ejection fraction, diabetes, randomized treatment strategy, previous heart failure, previous MI, smoking status, low-density lipoprotein cholesterol, and extent of myocardial ischemia. Abbreviations: HR, hazard ratio; CI, confidence interval. *Type 4a MI n = 31; ^#^Type 4a MI n = 24; ^§^Type 4a MI n = 110; ^^^Type 4a MI n = 85.

## 7. Predictors of PMI and Pharmacologic Strategies for its Reduction

Predictors of PMI include both anatomic, procedural and clinical variables [14]. Side-branch occlusion, multivessel disease, bifurcation lesions, long lesions, number of implanted stents, left anterior descending artery disease or left main disease are frequently associated with the occurrence of PMI. Older patients of female sex and with diabetes mellitus, renal dysfunction are more vulnerable to this complication.

Some pharmacologic strategies have been found to reduce PMI including statins, antithrombotic and antiplatelet agents. Small studies have shown that the administration of statins before PCI may reduce PMI rates compared with no treatment [45, 46]. The beneficial action of statins is likely mediated by an anti-inflammatory action rather than by their lipid-lowering effects, as demonstrated by their higher benefit among patients with elevated hs–C-reactive protein [47]. Another strategy targeting the ischemia/reperfusion injury pathogenesis of PMI which was explored in clinical trials was the remote ischemic myocardial conditioning, that can reduce the incidence of PMI, as well as the post-PCI release of troponin [48].

Antiplatelet therapy represents an effective pharmacologic treatment to reduce PMI. The first demonstration of a benefit came from the use of intravenous glycoprotein (GP) IIb/IIIa inhibitors, particularly abciximab, a Fab fragment of a chimeric human-murine monoclonal antibody. The *Evaluation of IIb/IIIa Inhibitor for Stenting* (EPISTENT) demonstrated that the use of this drug reduces periprocedural CK-MB elevations and possibly mortality after stenting [49]. The benefits of abciximab may be due in part to improvements in perfusion of the distal microvasculature but may also be secondary to its anti-inflammatory effects, through binding to the vitronectin receptor on endothelial, smooth muscle, and inflammatory cells [50] and to the Mac-1 integrin found on monocytes and neutrophils, responsible for the inflammatory response to vessel injury [51]. The use of GPIIb/IIIa inhibitors has progressively declined over the years, due to their propensity to increase bleeding and to the growing use of potent P2Y12 inhibitors.

Dual antiplatelet therapy with adequate preloading has resulted in significant reduction in PMI. Clopidogrel 600 mg loading dose at least 2 hours before intervention reduced PMI by 20 per cent compared with placebo [52], whereas prasugrel in the TRITON TIMI-38 trial decreased PMI by 30% in comparison with clopidogrel in ACS patients [53], particularly in those presenting with non-ST segment elevation [54]. Compared with clopidogrel, cangrelor (an intravenous P2Y12 inhibitor) significantly reduced cardiac events and MI occurring in the first 2 hours after randomization in patients treated with PCI enrolled in the CHAMPION-PHOENIX trial, supporting the importance of potent platelet inhibition in this setting [55].

Recently, evidence has been enriched from the results of the ALPHEUS trial, aimed at assessing if ticagrelor, a potent non-thienopyridine P2Y12 inhibitor, was superior to clopidogrel in reducing PMI in CCS patients undergoing high-risk elective PCI. This study failed to show any significant difference between the two pharmacologic treatments, despite a higher level of platelet inhibition achieved with ticagrelor compared with clopidogrel [56]. Similar data in CCS patients were observed with prasugrel versus clopidogrel in the SASSICAIA trial, which was however underpowered to show any difference between the two drugs [57]. The results of the ALPHEUS trial showing that early ischaemic events after elective PCI are not improved by more effective P2Y12 inhibition than achieved with clopidogrel may reflect non platelet-related pathogenetic mechanisms [58, 59]. It is likely, however, that potent P2Y12 inhibition is needed to reduce PCI-related events only in ACS patients, in whom thrombotic complications are frequent, but it is not necessary in those with stable coronary artery disease. Other potential explanation for the ALPHEUS data may be related to the methodology used in that trial to assess PMI, that was based on the third UDMI (troponin increases 5× URL associated with ancillary criteria, see Table 1) also including major myocardial injury in the primary outcome. It is possible that troponin increases of small entity after PCI may simply reflect baseline risk, atherosclerotic burden and procedural complexity.

## 8. Conclusions 

Periprocedural myocardial infarctions are usually secondary to angiographically visible flow-limiting complications. However lesion complexity and extent of coronary disease also correlate with biomarkers release after elective PCI, suggesting that this phenomenon may simply be a marker of baseline risk, atherosclerotic burden and procedural complexity [13]. These data call for caution in considering “stand alone” biomarkers elevations as indicative of procedural complications or to use them as a quality metric for PCI. Several definitions of PMI have been proposed, using different thresholds for either c-Tn or CK-MB increase, associated or not with ancillary criteria indicating new evidence of myocardial ischemia. The incidence of PMI greatly varies according to the different criteria used for the diagnosis (from 2% to 18%) with dramatic variations of trial results using different definitions [40, 41].

There is consensus that the most appropriate definition of PMI should have a significant impact on subsequent cardiovascular death. Since occurrence of PMI is added to spontaneous MI in the computation of long-term events it seems reasonable to use a definition of PMI that has a similar effect on cardiovascular mortality as that caused by spontaneous MI. A recent analysis in a large patients dataset shows that definitions using higher thresholds for cTn increase after PCI are more prognostic for cardiac mortality at 1 year compared with definitions based on lower cTn thresholds and are therefore preferable in daily practice and for interpretation of clinical trials [30].
